# 

**Published:** 1910-11-12

**Authors:** 


					THE SCIENTIFIC PRESS PUBLICATIONS.
A LEGAL HANDBOOK FOR THE USE
OF HOSPITAL AUTHORITIES.
By LEONARD TYER BRI3TOWE, Barrister-at-Law.
2/6 post free.
MEDICAL HISTORY FROM THE EARLIEST TIMES.
By E. T. WITH1NGTON, M.A.
Over 400 pp. Illustrated, 12/6 net, 12/10 post free.
HOSPITAL EXPENDITURE:
THE COMMISSARIAT.
For tlis Managers of Hospitals. 2/6 post free.
PHOTO-MICROGRAPHY.
By EDMUND J. SPITTA, M.R.O.S., F.R.A.S.
Demy 4to. with 41 Half-Tone Reproductions irom original negatives and
63 Illustrations in the Text, 12/-.
THE SCIENTIFIC PRESS, 28 & 29 Southampton Street. Strand. W.C.
The FUTURE of the HOSPITALS.
Medical Relief for All according to means.
A Scheme of Co-operation between the PATIENTS, the
HOSPITALS, and the STATE.
By Sir HENRY BURDETT, K.C.B., K.C.V.O.
Price 2d.
THE SCIENTIFIC PRESS, LTD.,
28 & 29 Southampton Street, Strand, London, W.C
And at Bookstalls,
Just Issued. Ninth Edition, Rewritten and Enlarged, 9s. net.
PHARMACY, MATERIA MEDICA & THERAPEUTICS.
Being a Commentary on the B.P. and its Drugs, with their Preparations,
and on all the new Non-offlcial Remedies in use, with Chapters on the
different processes used in Dispensing, Prescription-writing, Latin
Glossary, <fcc. By Sir W. WHITLA, M.D., LL.D., Professor, Materia
Medica, Queen's University, Belfast, and Senior Physician, Royal Victoria
Hospital.
By the same Author. New Work. 1,900 pages. Crown 8vo. 25s. net.
PRACTICE OF MEDICINE.
Containing in two Handy Volumes a complete and independent Article on
the Etiology, Symptoms, and Diagnosis of every Medical Disease; arranged
in Dictionary Form for Practitioners and Students.
London: Bailliere, Tindall & Cox, 8 Henrietta Street, W.O.
A MANUAL OF
MEDICAL EXERCISES.
By PERCY LEWIS, M.D., M.R.C.S.,
Hon. Medical Officer to Victoria Hospital, Folkestone.
66 pages, with 35 Illustrations, cloth, Is. 6d. net; post free, Is. 7d
H. K. LEWIS, 136 GOWER STREET, LONDON, W.C.
IMPORTANT TO HOSPITAL
SECRETARIES?
ANALYSIS LEDGER.
Specially Ruled In accordance with, and adapted to tbe
requirements of, the New Index of Classification recently
adopted by the Kins: Edward's Hospital Pund for
London, and which came into operation last year.
The Ledger is bound in half-basil, green cloth sides, printed
and ruled in best style on good paper, and is in eveiy
way a serviceable book for Hospital use.
Price ?1 12s. 6d.
Carriage extra.
THE SCIENTIFIC PRESS, LTD.,
28 k 29 Southampton St., Strand, London, W.O.
Florence has recently devised and recommended
a new reagent for testing for both bile and blood in
urine. The solution keeps well, and is made up
as follows: Pyridine, 50; alcohol, 50; chloroform,
50; and zinc acetate, 7.5 parts by weight.
When used, two or three cub. cm. of the urine and
twice as much of the reagent are mixed up together
in a test-tube. On standing the lower layer will be
colourless in the absence of bile pigments or blood.
If urobilin is present it will show a fine green fluore-
scence. If biliverdin is present it is green at first,
and also slowly develops fluorescence. Blood pig-
ment gives it a tint varying from pink to cherry red.
The intensity of the colour is, broadly speaking,
quantitative. The solutions give very clear spectro-
scopic bands.
Appointments and Resignations,
Mr. Archibald Cuff, F.R.C.S., has been appointed
Lecturer on Practical Surgery at the University of Shef-
field.
The newly appointed senior house surgeon at Beckett
Hospital, Barnsley, is Mr. S. H. Ward, M.R.C.S.,
L.R.C.P., late of London Hospital, at present housa-
surgeon at the General Hospital, Stroud.
Obituary.
The death is announced, suddenly, of Mr. William
Thomas Marston Clark, at the age of fifty-three. He
studied at St. Bartholomew's Hospital, took his M.R.C.S.
in 1879, his L.R.C.P. in 1880, and D.P.H. in 1893. His
appointments included those of surgeon to St. John's
Hospital, Twickenham, to the Twickenham Dispensary,
Lying-in Charity, and Boys' Home. He was also M.O.H.
and Sch.M.O. at Twickenham.
The death is reported of Dr. Patrick J. Fagan..
F.R.C.S.I., which occurred suddenly at his residence i?i
Dublin, at the early age of forty-seven. Born at Trim,
in the County Meath, he was educated at Castleknock
College and at the Catholic University Medical School,
to the staff of which latter institution he became attached
subsequently. He had for several years been on the
visiting staff of St. Vincent's Hospital, where he wa?
appreciated alike by colleagues and students for his learn-
ing, skill, and kindliness.
The Rector of Trowbridge has been re-elected President
of the Trowbridge Cottage Hospital, of which Mr. W. A,
Gouldsmith and Mr. J. D. Gouldsmith are the new Vice-
Presidents. Mr. F. R. Willis, hon. treasurer, and Mr,
W. J. Mann, hon. secretary, were also re-elected.
Elections and Appointments.
Miss Florence Reeves, who was trained at the London,
and has been sister at the Metropolitan Hospital, has beea
appointed matron of the Royal Liverpool Country Hospital
for Children.
General Peters has been elected President, and Admiral
White Vice-President, of the West of England Eye In-
firmary, of which Mr. Thomas Snow has been re-elected
Hon. Treasurer.
Sir Henry Mildmay has become the first president of
the new Odiham Cottage Hospital, Hampshire. Two
vice-presidents have been appointed in the persons of Lady
Petre and Miss' Mclntyre.
Mr. G. W. Mumford, of Liverpool, has been elected
President of the Alston Cottage Hospital. Mr. R. Elliot
and Mr. W. Sykes have been elected treasurers. The nevr
secretary is Mr. G. T. Elliot.
A cottage hospital is to be built at Oakham itt
memory of Mr. George Henry Finch, for many yeara
M.P. for Rutland and at the time of his death "Father"-
of the House of Commons. Sir Arthur Fludyer is
chairman of the committee, and tho sum of ?1,500 has
already been subscribed..
'214 THE HOSPITAL November 12, 1910.
Gifts anp Bequests.
r The Treasurer of the Leicester Infirmary has received a
legacy of ?20 under the will of the late Mr. George Wester-
man, of Coalville.
The Leicester Homecomers Festivities.?The Com-
mittee of the above Fund (the Mayor of Leicester, Coun-
cillor G. Chitham, Treasurer) has about ?50 in hand after
payment of outgoings, and this sum has been allocated as
follows : The Leicester Infirmary ?19, the Leicester Homes
for the Blind ?19, and ?10 to the Maternity Hospital.
Major Andrew Coats, D.S.O., has generously con-
, tributed ?100 to the reconstruction scheme and become an
rannual subscriber of ?20 to the Leicester Infirmary.
The erection of new buildings for the Nottingham and
?Midland Eye Infirmary having been decided upon, an
anonymous gift of ?1,000 from a friend is announced.
Mrs. Maria Corbould, of Somers Road, Reigate,
Surrey, widow of Dr. F. J. Corbould, who left estate
valued at ?57,435 net, has left ?200 to the Reigate and
Redhill Hospital.
The Right. Hon. Hedges Eyre Chatterton, P.C., of
? New Park, Blackrock, co. Dublin, for thirty-seven
years Vice-Chancellor of Ireland, formerly Attorney-
General for Ireland, has left ?200 to Sir Patrick Dun's
Hospital.
The executors of the late Alderman George Peters, a
former Mayor of Gloucester, have forwarded cheques for
?5,000 each to the Gloucestershire Royal Infirmary and the
Gloucester Children's Hospital on account of the share of
the residue to which those institutions are entitled under
his will.
The Court of Common Council has voted ?157 10s. to
the London Fever Hospital, ?105 to Chelsea Hospital for
Women, ?78 15s. to the " Friedenheim" Hospital,
?52 10s. for the Farringdon General Dispensary, ?21
for the Brixton Infirmary, and ?52 10s. for the Ber-
mondsey Medical Mission.
Miss Harriet Franks Davis, of Bridge Green (near
Tunbridge Wells), Sussex, has left ?250 to the Eye and
Ear Hospital, Tunbridge Wells, and ?100 each to the
British Medical Benevolent Fund, for increasing the
^annuities of the pensioners, and the Surgical Aid Society
(Tunbridge Wells branch).
Mr. John William Cadwalladr, of Swansea, has left
?100 each to the Swansea Hospital and to the vicar of
Swansea upon trust to expend the income therefrom annu-
ally on Empire Day on refreshments for the children of the
National and Parochial schools in memory of the "old
Master."
The treasurer of St. Bartholomew's Hospital has re-
.ceived the following further contributions towards the
special fund?namely, Martin's Bank, Ltd., ?52 10s. ;
Mr. H. Siccama, ?10; Miss R. Baker, ?10; the Misses
Trewby, ?10; the Salters' Company ?210, being the third
instalment of a grant of 1,000 guineas voted to the
hospital.
Prince Alexander George of Teck has received ?4,600
for the Prince Francis of Teck Memorial Fund in aid of
.'the Middlesex Hospital. The latest donations received
vinclude ?250 from the Society of Motor Manufacturers
and Traders,-?200 from Messrs. A. Cartier and Sons,
e?75 from Mr. and Mrs. Charles Davis, 50 guineas from
Mr. C. Brinsley Marlay, ?50 each from Lady Bathurst
and Mrs. E. Soutter, and ?20 from Mr. Greville Douglas.
Jottings.
The new cottage hospital at Abergeldy, which contains
four wards, of two beds each, and has cost ?2,300, was
opened free of debt a few days ago by Mr. Donald
Mirrilees, Garth, grandson of the late Sir Donald Currie.
It was decided at a county meeting held at Maidstone
last week to enlarge and endow the West Kent General
Hospital as a memorial to King Edward. It is hoped to
raise ?20,000. Towards this sum Mr. H. G. Kleinworth
has promised ?2,000 and Sir Marcus Samuel ?1,000.
The restored chapel of the Kent County Ophthalmic
Hospital at Maidstone wae dedicated last week, in the
presence of the board of management and medical staff,
by the vicar of Maidstone and the chaplain. Two
stained-glass windows, representing "The Blind Man"
and "Malchus," were unveiled.
At a representative conference of matrons of hospitals
called by the National Food Reform Association, held
at the Caxton Hall last Saturday, to discuss the feeding
of nurses, Miss It. Cox Daviea (Royal Free Hospital)
described a scheme by which her hospital was regularly
supplied with fresh garden produce. Daily hampers were
sent by a number of country friends with large estates
in rotation, and the scheme, which had been in operation
for four years, worked admirably.
Prince Alexander of Teck, accompanied by Princess-
Alexander of Teck, attended the meeting of the Middlesex
Hospital weekly board of governors last we6k, and while
he was thus engaged her Royal Highness was conducted
through the wards and various departments of the hos-
pital and the Cancer Charity by the lady superintendent,
Miss A. Lloyd Still. His Serene Highness left for
luncheon, and returned in the afternoon to continue the
work of the Prince Francis of Teck Memorial Fund.
Mrs. Kendal and Miss Lillie Burgess entertained the
patients of the Royal Hospital for Incurables, Putney
Heath, with a recital in the Assembly Hall last week.
Besides the patients, there was a large number of visitors,
as well as the following members of the board : Sir
William Clerke, Mr. Thomas W. Wickham (deputy chair-
man), Captain T. Clarence E. Goff, L.C.C., Mr. J. T. Bel-
frage, and Mr. T. Turton Wright. At the close Mrs.
Kendal thanked the patients for the enthusiastic recep-
tion given to her and Miss Burgess, and said she was
willing to give another recital at any time. She was pre-
sented with a bouquet by the matron, Miss Lucy S. Begg.
The Worcester memorial is to take the form of an addi-
tion to the Worcester General Infirmary, to be known as
the "King Edward VII. Annexe, for the more efficient
treatment of children and out-patients." The infirmary
has a special claim on the support of the city, inasmuch
as out of 1,800 patients 1,400 were from the city. When
Sir Henry Burdett visited the infirmary he pointed out
many points in which the building was no longer up to
date, and this proposed addition will help to remove that
reproach. It was announced that the patients and nurses
would hand over ?68, which they had collected for the
purpose of providing additional accommodation for
children, to the memorial fund.
TO HOSPITAL SECRETARIES AND OTHERS.
We invite contributions from any of our readers, and
shall be glad to pay, according to length, for items of news
for the institutional section (including appointments, resig-
nations, gifts, etc.) which we may publish. All payments
for manuscripts used are made as early as possible after
the beginning of each quarter. ^
GENERAL PRACTITIONERS'
CONTRIBUTIONS.
The Hospital devotes a special page to General
Practitioners' contributions. We therefore invite
from practitioners contributions based upon their
experience in the management of cases, and in the
treatment and diagnosis of disease; especially shall
we be prepared to welcome articles dealing, practi-
cally, with treatment, and with the use and value
of new remedies and methods.
No article should exceed 1,100 words, and, if
accepted, one guinea for a contribution of this
length will be paid to the writer after publication.
Each communication should be accompanied by a
stamped directed envelope for the return of the MS.
if found unsuitable.
Contributions should be written, or preferably
typed, on one side of the paper only, and all articles
sent in are accepted upon the distinct understanding
that thev are forwarded to The Hospital only.
Correspondence.
Correspondence on all subjects is invited, but no
communication can be entertained if the name and
address of the correspondent are not given as a
guarantee of good faith, but not necessarily for pub-
lication. All correspondents should write on one
side of the paper only.
The Relaxations of Medical Men.
We shall also be glad to pay for accepted contri-
butions, from any member of the profession, on the
subject of the relaxations of practitioners. This
opens up a wide field, as it includes natural history,
photography, sport, indoor recreations, and motor-
ing. Whenever possible, original illustrations and
photographs should be sent with the MS.
BUSINESS NOTICES.
Annual Subscriptions.
The rate of annual subscriptions to The
Hospital, either direct from the Office or through
agents, is: ?
For the United Kingdom  15s. a year.
For the Colonies and Abroad ... 17s. ,,
For the United Kingdom (with
Insurance Policy)  ?1 ,,
inclusive of postage in each case.
Letters relating to the Publishing, Sale and
Advertisement Departments must be addressed to
the Manager (not to the Editor): ?
The Manager,
The Hospital Building,
28 and 29 Southampton Street,
Strand, London, W.C.
THE
POCKET-BOOK of TEMPERATURE CHARTS.
Containing seventeen 12-hour and eight 4-hour Charts, perforated so that each Chart may be
easily removed after use.
Bound in paper cover. Suitable size for the pocket.
Price 6d. net; by post, 7d.
Of all Booksellers, or of
THE SCIENTIFIC PRESS, LIMITED, 28 & 29 Southampton Street, Strand, London, W.C.

				

